# The intensity of free radical processes and chaperone activity in the saliva of patients with type 2 diabetes

**DOI:** 10.37796/2211-8039.1407

**Published:** 2023-06-01

**Authors:** Anna A. Cheprasova, Sergey S. Popov, Alexander N. Pashkov, Aleksei N. Verevkin, Valentina O. Mittova, Konstantin K. Shulgin

**Affiliations:** a Department of Biology, Voronezh State Medical University named after N.N. Burdenko, Voronezh, Russian Federation; b Department of Organization of Pharmaceutical Business, Clinical Pharmacy and Pharmacognosy, Voronezh State Medical University named after N.N. Burdenko, Voronezh, Russian Federation; c Department of Medical Biochemistry and Microbiology, Voronezh State University, Voronezh, Russian Federation; d Teaching University Geomedi, LLC, Georgia

**Keywords:** 8-hydroxy-2′-deoxyguanosine, Chaperone activity, Oxidative stress, Saliva, Type 2 diabetes mellitus

## Abstract

**Introduction:**

Saliva is a clinically informative biological fluid that contains many biomarkers, allowing multiple analyses to be performed.

**Aim:**

The objectives of this study were the assessment of the serum and saliva levels of biochemical parameters and intensity of free radical processes in T2DM patients and the identification of the correlation between certain criteria.

**Methods:**

This case-control study included 40 T2DM patients, which were compared with 40 healthy individuals. The levels of glucose, cholesterol triglycerides, total protein, diene conjugates, and chaperone activity were measured using the spectrophotometric method. The concentration of 8-oxo-2′-deoxyguanosine was assessed by competitive enzyme-linked immunosorbent assay.

**Results:**

It was established that the progression of diabetes led to an increase in glucose in saliva. The content of 8-oxo-2′-deoxyguanosine and conjugated dienes increased in serum and this increase was associated with the level of glucose and glycated hemoglobin. The level of protein and chaperone activity increased in the saliva of patients with T2DM compared with the control. The correlation analysis revealed a relationship between total protein concentration and conjugated dienes and between chaperone activity and conjugated dienes in saliva.

**Conclusions:**

According to the results of the analysis, the pathological changes in DM affected the salivary glands and their secretions. The obtained results allowed us to recommend using saliva as an alternative to blood for the diagnosis and monitoring of T2DM treatments since it is readily available and quickly responds to changes in metabolism in the body.

## 1. Introduction

Diabetes mellitus (DM) is one of the most common diseases affecting more than 463 million people in the world [1]. In the Russian Federation, as in all countries of the world, there is also a significant increase in patients with diabetes. According to the federal register of diabetes in the Russian Federation at the beginning of 2020, 4.76 million patients with diabetes were identified: type 1 diabetes - 5.6% (261.3 thousand), type 2 diabetes (T2DM) - 92.4% (4.40 million), other types of DM - 2% (97.1 thousand). However, only identified and registered cases of the disease are taken into account and according to the results of a large-scale Russian epidemiological study (NATION), it is known that only 54% of cases of type 2 diabetes are diagnosed [2]. Thus, DM can be detected much later, when complications such as retinopathy, nephropathy, and damage to the great vessels of the heart, brain, and arteries of the lower extremities occur due to chronic hyperglycemia, which is the main cause of disability and mortality in patients with diabetes [3]. As a result, diabetes is the most important medical and social problem on a global scale [4]. Currently, there is a need to develop programs for the active detection of diabetes during the early stages of the disease [5,6].

Diagnosis of diabetes, as well as regular monitoring of compensation for the disease, is accompanied by constant blood sampling for analysis. Now, the search for new non-invasive methods for detecting diabetes is being carried out. Saliva is one of the promising targets for diagnosing DM.

Saliva is an accessible biomaterial providing a link between the external and internal environment of the body. The salivary glands selectively carry out the transport of substances from the blood plasma into the saliva, have an endocrine function, and respond to any changes in the state of internal organs and body systems [7].

Many researchers believe that a change in the composition of the saliva of the large salivary glands can be a marker of a pathological process in the body [8]. Metabolic disorders in T2DM patients are reflected in changes in the biochemical parameters of blood serum and mixed saliva [9].

Human saliva is a unique biological fluid, the change in biochemical parameters of which reflects the state of metabolic processes in the body and has clinical and diagnostic value [10]. The use of saliva for diagnostic purposes has several advantages: the collection of saliva is not invasive, it is atraumatic and painless, which allows multiple sampling of this biological fluid [11]. The composition of saliva is characterized by both organic and inorganic components. In the sediment of mixed saliva, there are mainly organic substances such as proteins, peptides, amino acids, and carbohydrates. The concentration of proteins in saliva is significantly lower than in blood plasma since blood proteins poorly pass through the blood-saliva barrier. In mixed saliva, up to 500 different proteins and polypeptides were detected, approximately 120–150 of which are secreted by the salivary glands.

Heat shock proteins (HSP) involved in the regulation of growth, development, signaling, cell death, and other physiological processes, were found in mixed saliva [12]. Expression of chaperones occurs as a result of the reaction of living systems to adverse environmental factors such as heat, hypoxia, ischemia, all types of radiation, hyper- and hypoosmolarity, oxidative stress, infections, heavy metals, and other stress factors [13]. An increase in the concentration of HSP in biological fluids and tissues of the body may be an indicator of the response to exogenous and endogenous damaging factors.

8-Oxo-2′-deoxyguanosine (8-oxo-dG) is a well-known biomarker of oxidative stress and associated diseases; it is determined in various tissues and body fluids: blood, urine, saliva, and liver [14]. Many studies indicate the possibility of using 8-oxo-dG as a biomarker of T2DM [15].

An increased concentration of glucose has a negative effect on the resistance of capillaries and increases the permeability of blood vessels in the oral cavity, which in turn affects the biochemical composition of saliva, in particular, contributes to the development of free radical oxidation, including lipid peroxidation (LPO) as a result of damage to biological membranes [16]. In this regard, the aim of the study was the assessment of the content of glucose, glycated hemoglobin, parameters reflecting the state of free radical homeostasis - the concentration of diene conjugates, 8-oxo-dG, and chaperone activity in saliva of healthy people and men and women with T2DM and the identification of the correlation between these criteria and some indicators of venous blood.

## 2. Methods

### 2.1. Study design and participants

This case–control study was conducted at the endocrinology department of the BUZ VO “Voronezh Regional Clinical Center for Specialized Types of Medical Care” Russian Federation. The diagnosis of T2DM was established in accordance with the classification criteria, according to clinical guidelines [1]. The research has been approved by the Ethics Committee of Voronezh State Medical University named after N.N. Burdenko (approval of the ethics committee of VSMU N. N. Burdenko for conducting clinical trial no. 1 from 27.02.2014). An informed consent form was signed by each participant before the research was conducted. All procedures performed in the study were in accordance with the 1964 Helsinki Declaration and Federal Law of the Russian Federation No. 323-FZ of November 21, 2011 “On the Basics of Health Protection of the Citizens in the Russian Federation.”

This study included 40 patients with T2DM (20 men and 20 women). The control group consisted of 40 apparently healthy individuals with normal indicators of general and biochemical blood tests. The exclusion criteria from the study were: periodontitis, viral hepatitis, acute infectious diseases, acute myocardial infarction, malignant neoplasms, and cerebrovascular accident.

### 2.2. The method for obtaining saliva samples

The collection of mixed saliva was carried out in the morning on an empty stomach after rinsing the mouth with water using a saliva collection kit (Sarstedt D-51588 Numbrecht), the cotton swab for saliva collection was kept in the mouth for 10 min. During the collection of saliva, the donors sat, did not speak, and breathed through their nose. A clear saliva sample for use in the study was obtained after centrifugation for 10 min at 1400 g.

### 2.3. Biochemical investigations

Quantitative determination of saliva glucose levels was performed by the glucose oxidase method. The absorbance measurements were carried out at 510 nm. The protein concentration of saliva samples was determined using the biuret reaction [17].

The chaperone activity of mixed saliva was determined using a model test system based on the suppression of insulin aggregation. The kinetics of insulin aggregation induced by dithiothreitol and a temperature of 40°C were measured by turbidimetric assay. Aggregation was recorded using a Spekol 100 spectrocolorimeter with a thermostated cuvette and an automatic recorder at a wavelength of 430 nm [18].

The determination of the concentration of 8-oxo-2′-deoxyguanosine in saliva was carried out by competitive enzyme-linked immunosorbent assay using an HT 8-oxo-dG ELISA Kit (Trevigen, Inc., USA); the registration was carried out at a wavelength of 450 nm.

Determination of the concentration of diene conjugates (DCs) in mixed saliva was performed using the spectrophotometric method based on the appearance of conjugated double bonds in polyunsaturated fatty acid molecules during LPO. The registration was performed at 233 nm.

The blood glucose level was assessed using a Satellite Plus glucometer (ELTA, Russia).

### 2.4. Statistical analysis

Statistical data processing was performed by the methods of mathematical and medical statistics using the Microsoft Office Excel data analysis package and the STADIA 7.0 statistical package (InCo, Russia). Quantitative data are presented as M ± m, with M− sample mean, m − standard error of the mean. For the identification of significant differences between independent groups, a Student’s t-test was used. For testing statistical hypotheses, a 5% significance level was used.

Pearson’s correlation coefficient was used to assess the correlation of the studied parameters of blood serum and mixed saliva.

## 3. Results

During the study, saliva and blood serum samples were obtained from 40 people with T2DM. The clinical parameters of study participants are presented in Table 1.

In the group of apparently healthy individuals, the glucose level in the blood serum was 4.3 mM in men and 4.1 mM in women, which correspond to acceptable values [1]. At the same time, the glucose concentration in patients with T2DM was significantly increased: in men by 2.2 times (p < 0.05), in women by 2.5 times (p < 0.05), compared with the indicators in the control group (Table 1).

There was also an increased content of glycated hemoglobin in the examined patients by 1.6 times compared to the control values (p < 0.05) in men, and by 1.7 times (p < 0.05) in women. The concentration of glucose in saliva in patients with T2DM compared with the control group was increased by 3.5 times (p < 0.05) in men and 3.4 times (p < 0.05) in women, which is consistent with the data on the degree of hyperglycemia in the blood (Table 1).

An increase in the concentration of cholesterol and triglycerides in both blood serum and saliva was revealed in patients with T2DM. At the same time, a positive correlation between the content of total cholesterol in saliva and blood serum was observed (patients with T2DM, men, r = 0.550, patients with T2DM, women, r = 0.831). A high positive correlation between the concentration of triglycerides in the blood serum and saliva in patients with T2DM was identified (men, r = 0.733, women, r = 0.826).

**Fig. 1:**
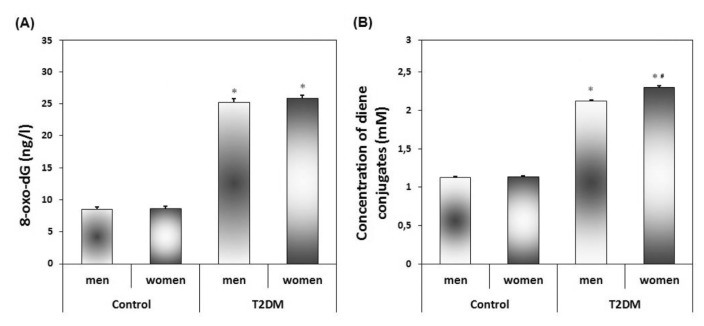
Concentration of 8-oxo-2′-deoxyguanosine (A) and diene conjugates (B) in saliva in the control group and the group of patients with type 2 diabetes mellitus. (M ± m). A single asterisk (*) indicates a statistical difference compared to the control group (p < 0.05). A pound sign (#) indicates a statistical difference between women and men in subgroups (p < 0.05).

The results of the study demonstrated a threefold increase in the content of 8-oxo-dG in the mixed saliva of patients with T2DM in both men and women in comparison with apparently healthy individuals (Fig. 1). The content of DC - the primary LPO products increased in the saliva of patients with T2DM by 1.8 times (p < 0.05) in men and by 2.0 times (p < 0.05) in women compared with the corresponding values in the control group.

The dependence of the intensity of free radical processes on the level of glycaemia was confirmed by the data of correlation analysis between the concentration of glucose in saliva and the concentration of DC in men (p_xy_ = 0.498) and women (p_xy_ = 0.387) with T2DM. Associations between the concentration of 8-oxo-dG and glycated hemoglobin (p_xy_ = 0.535) and between the content of 8-oxo-dG and the concentration of glucose in the blood (p_xy_ = 0.534) were also detected.

As our studies have shown, the concentration of total protein in the saliva of patients with T2DM was increased on average by 29.4% (p < 0.05) in men and by 33.4% (p < 0.05) in women compared with the control group (Table 1). An increase in chaperone activity in saliva was also revealed in patients with T2DM: in men - by 1.9 times (p < 0.05), in women - by 2.1 times (p < 0.05) compared with healthy donors. Significant differences between the chaperone activity of saliva in male and female patients with T2DM were revealed (p < 0.05). Thus, correlations between the concentrations of total protein and DC in saliva in men with T2DM (p_xy_ = 0.347) and between the concentration of DC and chaperone activity of saliva in both men (p_xy_ = 0.313) and women (p_xy_ = 0.327) with T2DM were found.

It should be noted that data reflecting the correlation relationship between the age of patients with T2DM and biochemical parameters in saliva were obtained. Thus, positive correlations were found between age and glucose (p_xy_ = 0.314) and DC concentrations (p_xy_ = 0.414) in women.

## 4. Discussion

The data obtained in the study indicate that the values of the biochemical parameters of blood and saliva significantly differ in patients from the control and experimental groups. In addition, the revealed correlations indicate a direct relationship between free radical processes and glucose levels in saliva of the patients with T2DM, which can lead to damage to the tissues of the oral cavity and cause concomitant diseases.

With diabetes, many metabolic processes, including the lipid metabolism, are impaired, which was expressed in an increase in the level of cholesterol and triglycerides in the blood serum of patients with T2DM. Probably, this was due to the increased level of lipid production in hepatocytes, leading to the excessive formation of very low-density lipoproteins. In addition, in patients with T2DM, the inability of insulin to inhibit the release of very low density lipoproteins from the liver was demonstrated [19]. The increase in the level of triglycerides and cholesterol in saliva correlated with the level of these metabolites in the blood serum in patients with the pathology and significantly differed from these parameters in the control group of patients. This finding indicates that the transport of the studied compounds into saliva is determined by their plasma level.

The activation of a large number of alternative pathways of glucose metabolism: sorbitol (polyol) and hexosamine pathways, autooxidation during DM is known. According to the literature, an increased glucose content in saliva leads to the accumulation of end products of glycation, contributing to an increase in the production of reactive molecules damaging biological molecules, which was reflected in an increase in the level of DC and 8-oxo-dG [20,21]. The interaction of end products of glycation with cell receptors of monocytes and macrophages also contributes to an increase in ROS production and enhanced oxidative stress [22].

It is known that most salivary proteins originate from the salivary glands, but in various diseases, including T2DM, they can have a different origin. Associated, for example, with an inflammatory process or tissue damage. Probably, developing oxidative stress is one of the factors involved in tissue damage, which was confirmed by correlation analysis. In addition, the end products of glycation formed during T2DM are able to interact with monocytes, promoting their activation, which is characterized by the induction of migration and the release of proinflammatory cytokines [23].

These cytokines promote tissue damage primarily by enhancing the inflammatory activation of T-cells as well as facilitating neutrophil degranulation. Probably, an increase in the concentration of total protein and chaperone activity in saliva in patients with T2DM was associated with hyperglycemia and the development of oxidative stress, which was confirmed by the results of correlation analysis.

The revealed correlations between the DC level and the chaperone activity of saliva were probably due to the activation of HSP synthesis under the influence of ROS [24]. In addition, it is known that at low concentrations of methylglyoxal (formed during glucose metabolism), a modification of certain residues of αA-crystallin occurs, which is accompanied by a change in the physicochemical properties and an increase in chaperone activity [25].

The analysis of the correlations between the age of patients and the biochemical parameters of saliva indicates that T2DM belongs to diseases associated with aging, in the pathogenesis of which the development of oxidative stress plays an important role.

## 5. Conclusions

The obtained results allowed us to recommend saliva as an alternative to blood for the diagnosis and monitoring of T2DM treatment, since this biological fluid is readily available and quickly responds to changes in metabolism in the body. The method of collecting saliva is painless and atraumatic, which helps to reduce the risk of infection for patients and medical personnel, and also allows multiple sampling for research.

## Figures and Tables

**Table 1:** Clinical and biochemical characteristics of the study subjects.

Clinical parameters	Control		T2DM	
	
	Subgroup 1 (*n* = 20)	Subgroup 2 (*n* = 20)	Subgroup 1 (*n* = 20)	Subgroup 2 (*n* = 20)
Age (years)	41.05 ± 3.54	43.35 ± 3.18	62.60 ± 1.92	62.85 ± 2.78
Gender	men	women	men	women
Diabetes duration, years			14.70 ± 1.75	12.10 ± 1.39
BMI (kg/m2)			33.6 ± 1.20	34.06 ± 0.96
		Serum		
Glucose (mM)	4.3 ± 0.13	4.2 ± 0.11	9.5 ± 0.53*	10.4 ± 0.75*
HbA1C (%)	4.8 ± 0.08	4.8 ± 0.08	7.7 ± 0.43*	8.1 ± 0.48*
Cholesterol (mM)	4.5 ± 0.08	4.5 ± 0.09	5.1 ± 0.18*	6.0 ± 0.28*
Triglycerides (mM)	1.0 ± 0.07	1.2 ± 0.05	1.9 ± 0.12*	2.3 ± 0.21*
		Saliva		
Glucose. mM	0.17 ± 0.01	0.17 ± 0.01	0.60 ± 0.01*	0.59 ± 0.01*
Cholesterol. mM	0.06 ± 0.01	0.06 ± 0.01	0.15 ± 0.01*	0.15 ± 0.01*#
Triglycerides. mM	0.05 ± 0.01	0.05 ± 0.01	0.18 ± 0.01*	0.19 ± 0.01*#
Total protein. g/l	3.77 ± 0.03	3.73 ± 0.03	4.88 ± 0.04*	4.97 ± 0.04*
Chaperone activity.	13.31 ± 0.63	12.82 ± 0.61	25.43 ± 0.36*	26.70 ± 0.37*#
% suppression of aggregation				

Values are expressed as mean ± standard deviations.

A single asterisk (*) indicates a statistical difference compared to the control group (p < 0.05).

A pound sign (#) indicates a statistical difference between women and men in subgroups (p < 0.05).
